# Genistein inhibits the growth and regulates the migration and invasion abilities of melanoma cells via the FAK/paxillin and MAPK pathways

**DOI:** 10.18632/oncotarget.15535

**Published:** 2017-02-20

**Authors:** Shuna Cui, Juan Wang, Qingqing Wu, Jing Qian, Changshui Yang, Ping Bo

**Affiliations:** ^1^ Jiangsu Key Laboratory of Integrated Traditional Chinese and Western Medicine for Prevention and Treatment of Senile Diseases, Medical College of Yangzhou University, Yangzhou, China; ^2^ Department of Gynaecology And Obstetrics Affiliated Hospital of Yangzhou University, Yangzhou, China; ^3^ Jiangsu Co-Innovation Center for Prevention and Control of Important Animal Infectious Diseases and Zoonoses, Yangzhou, China

**Keywords:** genistein, melanoma cells, invasion and migration, FAK/paxillin pathway, MAPK pathway

## Abstract

Genistein is one of the main components of soy-based foods, which are widely known for their many benefits, including anti-cancer, anti-inflammatory, and antioxidant effects. In this study, we investigated the anti-metastasis effects of genistein on B16F10 melanoma cells. Our results showed that genistein strongly inhibited B16F10 cell proliferation and induced apoptosis in time- and concentration-dependent manners. Genistein altered the morphology of B16F10 cells to an elongated shape with slim pseudopodia-like protrusions. Moreover, genistein inhibited the invasion and migration abilities of B16F10 cells in a dose-dependent manner. On one hand, a high concentration of genistein (100 μM) significantly inhibited cell adhesion and migration, as shown by wound healing assays and transwell-migration and invasion assays. Furthermore, the expression levels of p-FAK, p-paxillin, tensin-2, vinculin, and α-actinin were decreased by genistein. As a result, genistein is believed to strongly downregulate the migration and invasion abilities of B16F10 cells via the FAK/paxillin pathway. Moreover, p-p38, p-ERK, and p-JNK levels were also dramatically decreased by treatment with genistein. Finally, genistein significantly decreased the gene expression of FAK, paxillin, vimentin, and epithelial-to-mesenchymal transition-related transcription factor Snail, as shown by real-time PCR (qPCR) analysis. On the other hand, a lower concentration of genistein (12.5 μM) significantly promoted both invasion and migration by activating the FAK/paxillin and MAPK signaling cascades. Taken together, this study showed for the first time that genistein exerts dual functional effects on melanoma cells. Our findings suggest that genistein regulates the FAK/paxillin and MAPK signaling pathways in a highly concentration-dependent manner. Patients with melanoma should therefore be cautious of consuming soy-based foods in their diets.

## INTRODUCTION

Melanomas are malignant skin tumors that have a poor cure rate because of their invasive behavior. Today, tyrosine kinase inhibitors and immunomodulating agents are widely used in clinical therapy. However, these agents are associated with high incidences of adverse and toxic effects [1, 2]. Therefore, new treatment approaches with lower toxicity and higher specificity to prevent the progression of melanomas cells are desperately needed.

Cancer metastasis, a complex process involving cell migration, adhesion, and invasiveness, is often associated with melanomas. Benign tumor cells acquire the ability to migrate and invade, which are important steps in their transformation into malignant cancer cells. Cell migration is a dynamic and multistep process involving leading edge protrusion, turnover of focal adhesions, generation of traction forces, and tail retraction and detachment [3, 4]. Cell invasion is a process that can be initiated by altered integrin surface expression and the release or activation of several enzymes that degrade the extracellular matrix (ECM), allowing detachment of the cells from the primary tumor and healthy tissue [5, 6]. Various types of highly mobile cancer cells share certain features, such as epithelial-to-mesenchymal cell transition, the formation of filopodia or lamellipodia, and changes in cytoskeleton structure and morphology [7–9].

Focal adhesion kinase (FAK) is an important intracellular tyrosine kinase that plays vital roles in the regulation of ECM integrin signaling, cell cycle progression, cell motility, cell migration, and cell survival [10, 11]. Increased FAK expression and tyrosine phosphorylation have been observed in many malignant human tumors, and elevated expression of FAK is correlated with poor prognosis [12–14]. The inhibition of FAK functionality by small molecules reduced both the motility and viability of cancer cells in both mouse models and human clinical trials [15–17], suggesting that FAK might be a potential therapeutic target to treat highly invasive types of cancers. The COOH-terminal domain of FAK interacts with integrin-associated proteins; for example, FAK is activated upon cell binding to ECM proteins and forms a transient signaling complex with the steroid receptor coactivator (Src) family of protein tyrosine kinases [18]. Both Src and paxillin are important ligands and downstream effectors of activated FAK. The phosphorylation of FAK^Tyr925^ by Src affects its complex formation with paxillin and vinculin and further activates downstream pathways such as the MAPK or Rho/Rac/Cdc42 cascades that regulate cellular morphology and motility [4, 14]. Moreover, the FAK/paxillin interaction is modulated by the cytoskeletal adapter protein vinculin via the extracellular signal-regulated kinase (ERK) pathway [19].

Paxillin is a cytoskeletal protein that colocalizes with FAK at focal adhesion contacts and is a downstream target of FAK [20]. Paxillin is highly expressed in many cancers and is tightly associated with the development, progression, invasion, metastasis, and poor prognosis of multiple cancers [21–24]. Currently, increasing evidence has demonstrated that the FAK/paxillin signaling pathway participates in the processes of cancer invasion and metastasis via different molecular mechanisms [25–27]. Disrupting the interaction between FAK and paxillin blocks the migration and invasion of cancer cells [22]. Therefore, FAK/paxillin pathway activation is recognized as a potential predictor of cancer metastasis.

The signaling mediated by mitogen-activated protein kinases (MAPKs) is one of the most important cellular mechanisms responsible for melanoma metastasis, as it promotes cell proliferation, survival, invasion, and tumor angiogenesis [28, 29]. Indeed, MAPK signaling cascades are highly activated in melanomas, making them an appealing target for therapies [29]. ERK1/2, the c-Jun amino-terminal kinase (JNK), p38 kinase, and ERK5 are the four alternative MAPKs activated by distinct stimuli. Among these four cascades, the RAS/RAF/MEK/ERK1/2 pathway has attracted the most attention due to its critical role in regulating cell proliferation and survival. Therefore, inhibition of either the FAK/paxillin or MAPK pathway in cancer cells could be an effective strategy for preventing cancer cell migration and invasion.

The epithelial-mesenchymal transition (EMT) is a complex of cellular and molecular processes by which epithelial cells acquire mesenchymal and migratory properties [30]. The increased expression levels of both mesenchymal (e.g., fibronectin, vimentin, N-cadherin, α-smooth muscle actin) and invasion markers are hallmarks of EMT [15]. Transcriptional repressors (Snail, Slug, Twist, or ZEB1/2) are involved in EMT during development and respond to EMT stimuli to repress E-cadherin expression during tumor progression [31]. Moreover, FAK plays a critical role in tumor-associated EMT by promoting intracellular signaling pathways, which leads to downregulation of E-cadherin to further allow tumor cell migration/invasion [15].

Genistein is a naturally occurring isoflavone found predominantly in soy products, which are highly consumed in Asia. This compound is bioactive, exhibiting anti-inflammatory properties and playing roles in tyrosine kinase inhibition and phytoestrogen and anti-cancer effects [32]. The inhibitory effects of genistein on carcinogenesis and cancer progression have been recognized for years. Experimental evidence has shown that the inhibition of cancer cell growth by genistein is mediated by the modulation of genes related to the control of cell cycle and apoptosis, invasion, and metastasis [33–35]. However, whether genistein affects the viability or motility of melanoma cells remains unclear. In the present study, we aimed to investigate the effects of genistein on melanoma cells. Our results demonstrate that genistein exerts a dual functional effect on the invasion and metastasis of melanoma cells in a concentration-dependent manner. Furthermore, we showed that the FAK/paxillin and MAPK signaling cascades might be potential targets of genistein.

## RESULTS

### Genistein inhibits the proliferation of melanoma cells

**Figure 1:**
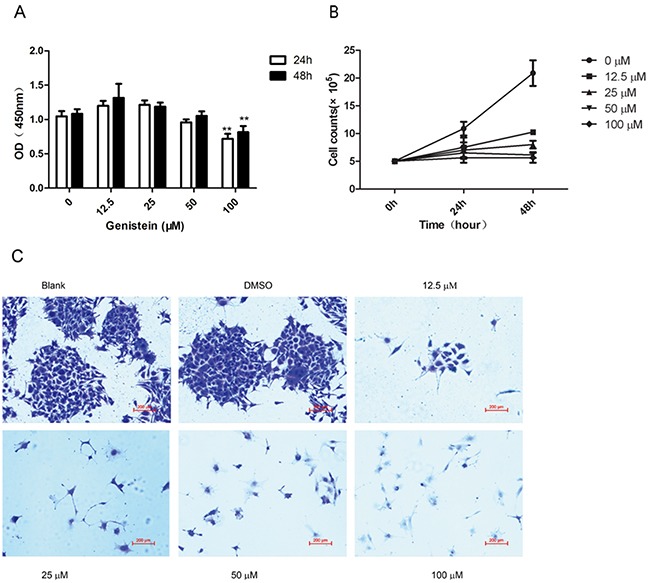
**A**. Effects of genistein on the viability of B16F10 cells as determined by WST-8 assay. B16F10 cells were incubated with different concentrations of genistein for 24 and 48 h. **B**. Cell numbers were counted in the 6-well plates after incubation with genistein for 24 and 48 h. **C**. Long-term colony formation assay of B16F10 cells after treatment with genistein (0, 12.5, 25, 50, and 100 μM). Cells were grown in the absence or presence of genistein at the indicated concentrations for 7 days. Cells were fixed and stained with crystal violet. Data shown are the averages of three replicates; the experiment was repeated three times. *p<0.05, **p<0.01, compared with the DMSO control. Scale bar is 200 μm.

We evaluated the influence of genistein, at various concentrations and incubation times, on the viability of melanoma cells using the WST-8 assay. The results showed that genistein has an inhibitory effect on cell viability in time- and dose-dependent manners (Figure 1A). We found that only the highest concentration of genistein evaluated (100 μM) significantly inhibited the growth of B16F10 cells after 24 and 48 h of incubation. In contrast, lower concentrations (12.5–50 μM) of genistein did not have affect cell viability. To confirm this result, we also counted the number of cells after 24 and 48 h of incubation with genistein. As shown in Figure 1B, genistein significantly decreased cell numbers by 24 h at concentrations of 50 and 100 μM. The effects were even more pronounced after 48 h (p<0.01). Furthermore, genistein (12.5–100 μM) showed a significant inhibitory effect on cell proliferation, compared with the control, in a colony forming assay (Figure 1C). These results demonstrate that genistein inhibits melanoma cell proliferation at concentrations of 12.5–100 μM.

### Genistein induces apoptosis of melanoma cells

**Figure 2:**
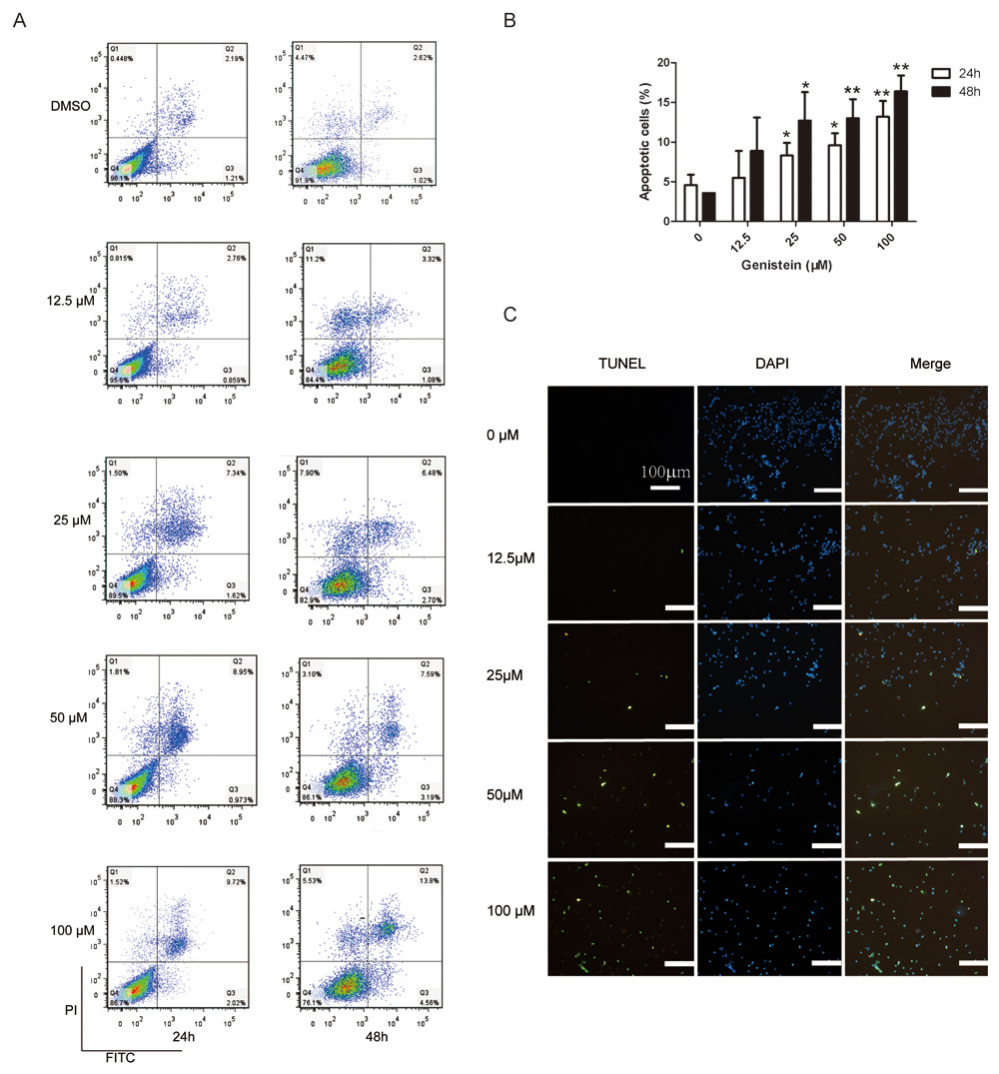
Flow cytometric analysis of B16F10 cell apoptosis after treatment with genistein for 24 and 48 h **A**. Influence of the genistein concentration on the ratio of apoptotic cells after treatment for 24 and 48 h. **B**. The experiment was repeated three times independently. Percentage of total apoptotic cells = Percentage of (Q2+Q3). In each experiment, 10,000 cells were counted (100%). **C**. TUNEL assay evaluating apoptosis after treatment with genistein for 48 h. Scale bar is 100 μm. *p<0.05, compared with DMSO control; **p<0.01, compared with DMSO control.

We further investigated whether genistein induces apoptosis of melanoma cells. As shown in Figure 2A–2B, at concentrations exceeding 50 μM, genistein significantly increased the number of apoptotic cells. Indeed, genistein (50–100 μM) induced DNA fragmentation after 24 and 48 h of treatment compared with the control (dimethyl sulfoxide [DMSO]) (Figure 2C).

### Genistein influences the morphology of melanoma cells

**Figure 3:**
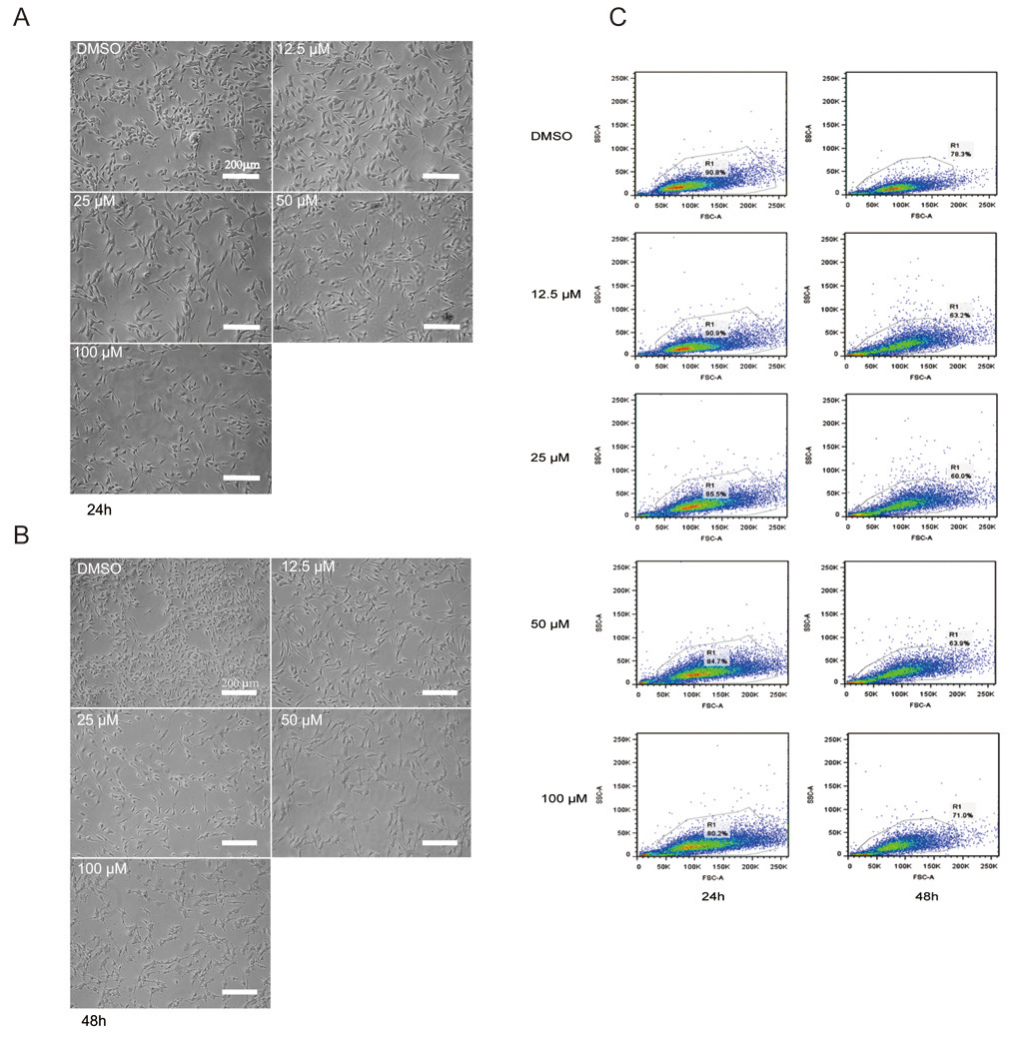
**A**. Morphological changes in B16F10 melanoma cells after treatment with genistein for 24 h. **B**. Morphological changes in B16F10 melanoma cells after treatment with genistein for 48 h. The pictures were taken under an inverted contrast microscope. Scale bar is 200 μm. **C**. Flow cytometric analysis of B16F10 cells treated with genistein for 24 and 48 h. Gating of the cells is shown in the plots of the forward versus side scatter of the cells.

Genistein treatment induced morphological changes in time- and dose-dependent manners (Figure 3). After 24 h, the control cells were confluent and spindle shaped, whereas the genistein-treated cells were well-attached, larger in size, and elongated with long and slim pseudopodia-like protrusions (Figure 3A). After 48 h, these morphological changes became more significant (Figure 3B). We confirmed these results using flow cytometry, analyzing the intensities of forward and side scatter light. As shown in Figure 3C, the cell populations with high side scatter intensities increased after treatment with 50–100 μM genistein for 48 h. In addition, the abundance of very small cells and cellular fragments (low forward and side scatter intensity values, respectively) increased with both the genistein concentration and incubation time, indicating the presence of apoptotic cells and cellular debris.

### Genistein influences the adhesion of melanoma cells

**Figure 4:**
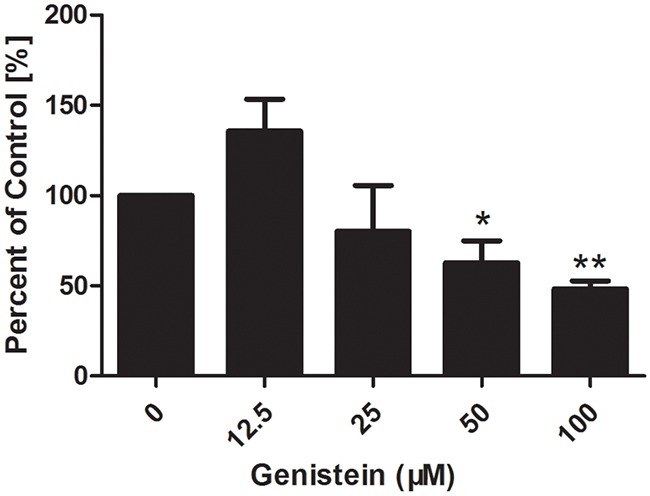
Genistein inhibits the adhesion of B16F10 melanoma cells Cells were incubated with genistein for 24 h. Unattached cells were first removed, and then attached cells were mixed in 4% paraformaldehyde and stained with crystal violet solution for 10 min at room temperature. The OD value was measured at 570 nm using a microplate reader. Percentage of adhered cells was calculated relative to the control cells. The experiment was repeated three times. *p<0.05, compared with DMSO control; ** p<0.01, compared with DMSO control.

Cell adhesion is an important step in cell invasion. As such, we investigated the effect of genistein on cell adhesion (Figure 4). Our data showed that pre-treatment of B16F10 cells with genistein 12.5, 25, 50 and 100 μM for 24 h significantly inhibited cell adhesion in a dose-dependent manner, showing inhibition levels of 135%, 80%, 62% and 48% compared with the control, respectively.

### Genistein influences cell migration

**Figure 5:**
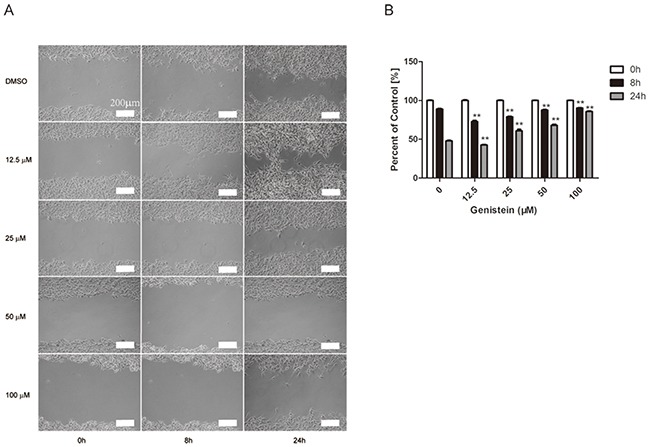
Genistein inhibits the mobility of B16F10 cells Cells were plated into a 6-well plate for confluent monolayer formation in complete medium. Cell monolayers were wounded using a sterile 200 μL micropipette tip, and the remaining cells were incubated in medium containing 0, 12.5, 25, 50, and 100 μM genistein for 24 h. At the indicated time (0, 8, and 24 h) after scraping, the wound areas were photographed **A**. and the percentage of cell migration inhibition **B**. was calculated as described in the Materials and Methods. Scale bar is 200 μm. The experiment was repeated three times. **p<0.01, compared with DMSO control for each time point.

A wound healing (cell migration) assay was performed to investigate whether genistein inhibits cell mobility. As shown in Figure 5, continuous migration of B16F10 cells was observed in the control group. Cell migration was significantly reduced in B16F10 cells treated with 50 and 100 μM genistein (p<0.01). Interestingly, cell migration was significantly enhanced after treatment with 12.5 μM genistein.

### Genistein influences cell migration and invasion

**Figure 6:**
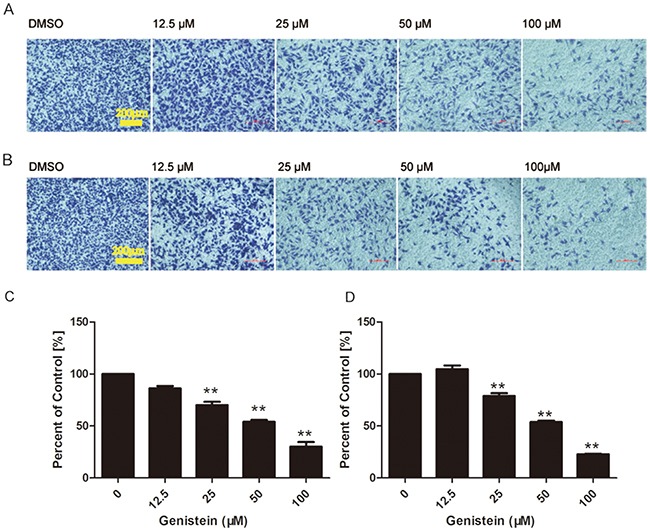
A higher concentration of genistein suppressed the migration and invasion of B16F10 cells *in vitro* Cells were seeded on membranes incubated with different concentrations of genistein for 24 h. Cells that crossed the membrane and cells on the surface of the lower side of the membrane were stained with crystal violet, photographed under a light microscope at a magnification of ×100, and **A**. counted **C**. Cells that penetrated through the Matrigel™ to the lower surface of the filter were stained with crystal violet, photographed under a light microscope at ×100, and **B**. counted **D**. Results were obtained from three independent experiments. **p< 0.01 compared with solvent control. Scale bar is 200 μm.

Cell migration and invasion play important roles in cancer metastasis. Thus, we measured the inhibitory effects of genistein on B16F10 cell migration and invasion by transwell assays. The treatment of B16F10 cells with increasing concentrations of genistein led to a dose-dependent decrease in vertical cell migration through the Transwell® filter. Genistein significantly inhibited *in vitro* cell migration by 30%, 54%, 70%, and 86%, at concentrations of 12.5, 25, 50 and 100 μM, respectively, at 24 h compared with the control cells (Figure 6A–6C), respectively. We obtained similar results in a wound healing assay, in which genistein inhibited the *in vitro* mobility of B16F10 cells (Figure 5).

Cell invasion was measured in modified Boyden chambers coated with Matrigel™, and the results are shown in Figure 6B–6D. The invasion of B16F10 cells was reduced by genistein treatment in a dose-dependent manner. Genistein significantly inhibited *in vitro* cell invasion by 22%, 53%, 78%, and 104% at 12.5, 25, 50 and 100 μM, respectively, at 24 h compared with the control cells. However, treatment with 12.5 μM genistein stimulated both cell migration and invasion, which coincides with the results from the wound healing assay (Figure 5).

### Genistein regulates the levels of FAK/paxillin and MAPK pathway proteins

**Figure 7:**
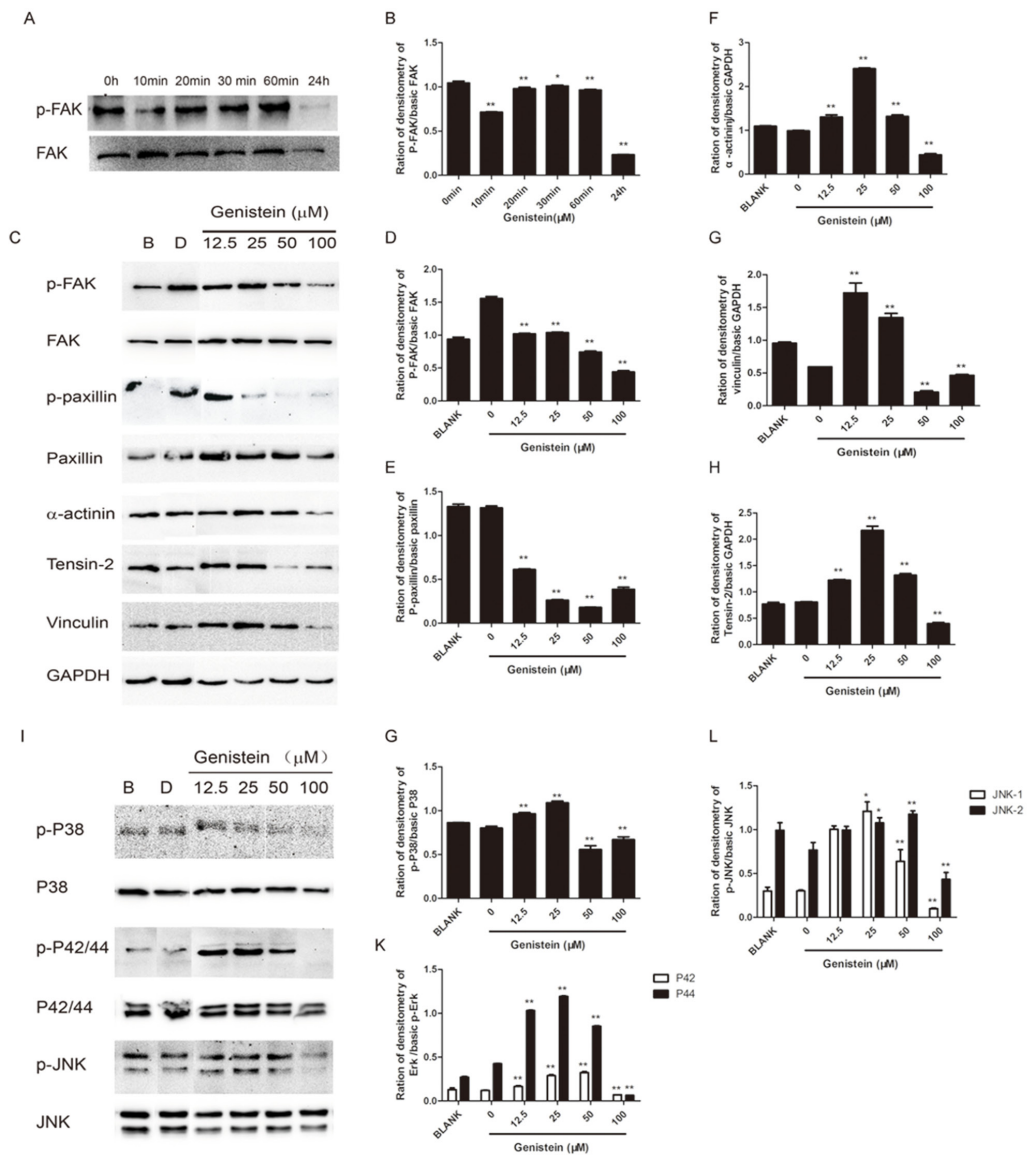
Genistein influences the expression of FAK/paxillin and MAPK pathway proteins in B16F10 cells **A**. Total cell lysates from B16F10 cells were prepared after treatment with genistein for 0, 10, 20, 30, and 60 min and 24 h. Next, 40 μg of each cell lysate were loaded onto the gel. (A) After blotting, the membranes were probed with p-FAK and FAK antibodies as described in the Materials and Methods section. **B**. Integrated band intensities as determined using Image J software. **C**. Total cell lysates from B16F10 cells were prepared after treatment with genistein for 24 h. Next, 40 μg of the cell lysates were loaded onto the gel. After blotting, the membranes were probed with antibodies against FAK/paxillin pathway proteins as described in the Materials and Methods section. **D-H**. Integrated intensity band intensities as determined using Image J software. **I**. The membranes were probed with MAPK pathway antibodies as described in the Materials and Methods section. **G-L**. Integrated band intensities as determined using Image J software. *p<0.05, **p<0.01, compared with solvent control.

We have shown that genistein inhibited the cell migration and invasion abilities of B16F10 cells. Both MAPK and FAK/paxillin pathways are associated with tumor progression, migration, invasion, and metastasis in many types of tumors [22, 28, 36]. Thus, we further investigated whether genistein inhibits cell migration and invasion via the suppression of proteins involved in the MAPK and FAK/paxillin pathways. The results from western blot analysis are shown in Figure 7. First, we performed time-course (0, 10, 20, 30, 60 min and 24 h) experiments to compare p-FAK^Tyr925^ and FAK levels after genistein treatment. Our results showed that genistein inhibited both p-FAK^Tyr925^ and FAK levels after treatment for 24 h (Figure 7A–7B). Furthermore, the incubation of B16F10 cells with genistein (24 h) inhibited the phosphorylation of both FAK and paxillin. The protein levels of α-actinin, vinculin, and tensin-2 were also strongly regulated by genistein in a concentration-dependent manner. Higher doses (50–100 μM) of genistein inhibited the expression of those proteins, whereas a lower dose (12.5 μM) enhanced their expression. These results indicate that genistein influences cell migration and invasion, possibly via regulation of the FAK/paxillin pathway (Figure 7C–7H).

**Figure 8:**
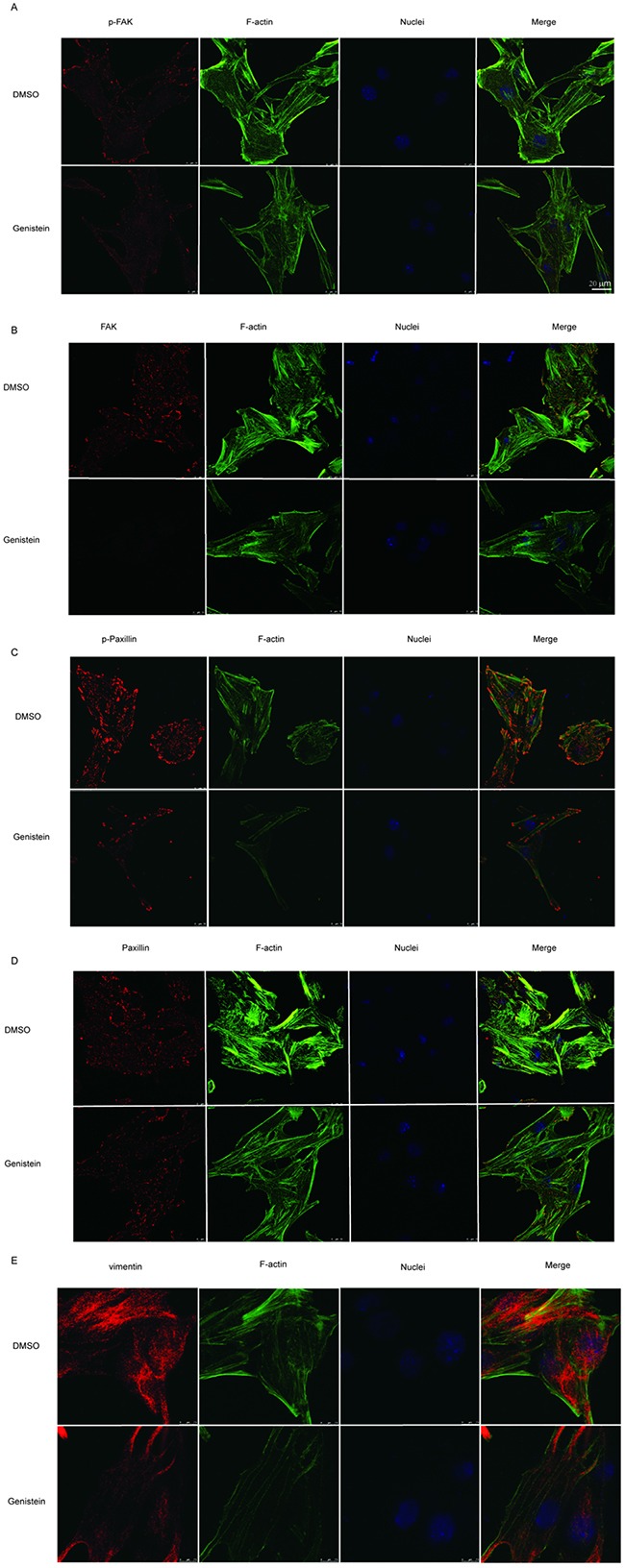
Modulation of p-FAK, FAK, p-paxillin, paxillin, and vimentin by genistein B16F10 cells were treated with or without 100 μM genistein for 24 h. The cells were then fixed and subjected to immunofluorescence staining with p-FAK, FAK, p-paxillin, paxillin, and vimentin primary antibodies, followed by secondary antibodies (red color), coupled with iFluor 488-conjugated Phalloidin (for F-actin, green color) and DAPI (for nucleus, blue color) staining. Scale bar is 20 μm.

We further investigated the influence of genistein on the MAPK pathway, demonstrating that treatment with 100 μM genistein strongly inhibited the phosphorylation of p38, p42/44, and p-JNK, whereas low doses (12.5–25 μM) stimulated the phosphorylation of JNK, p38, and p42/44 (Figure 7I–7L). The results of immunofluorescence staining showed that genistein altered the structure of F-actin filaments, reduced the levels of phospho-FAK/FAK and phospho-paxillin/paxillin around the periphery of cell membranes, and decreased the expression of the EMT marker vimentin (Figure 8).

### Genistein alters the gene expression of FAK, paxillin, vimentin, and Snail

**Figure 9:**
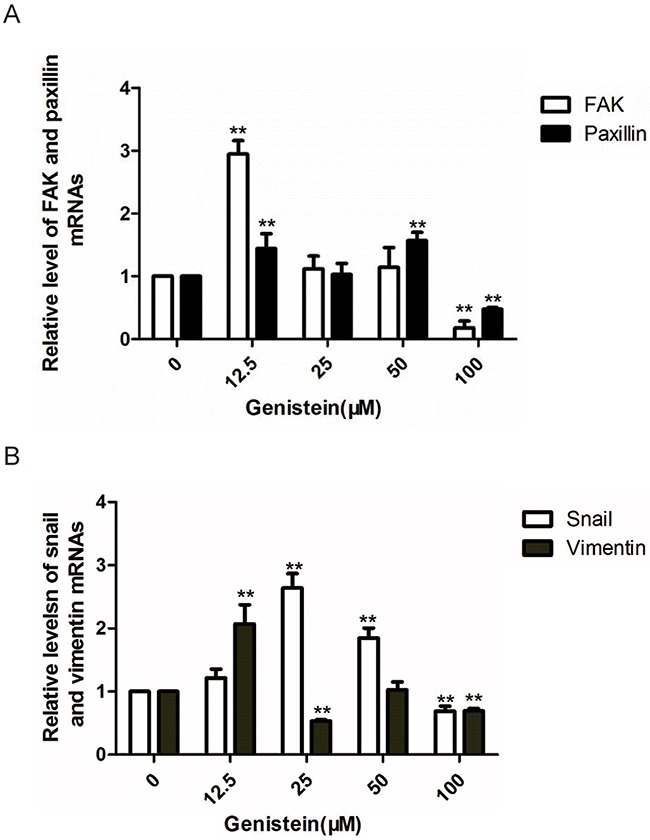
Genistein inhibits the relative mRNA levels of FAK, paxillin,vimentin, and Snail in B16F10 cells (as determined by RT-PCR) GAPDH were utilized as internal controls, and the relative mRNA levels were calculated according to the 2^−△△Ct^ method. *p<0.05, **p<0.01, compared with solvent control.

Because we observed that genistein regulated the expression of proteins in the FAK/paxillin pathway, we also measured the gene expression of both FAK and paxillin. We found that treatment with 100 μM genistein significantly downregulated FAK and paxillin mRNA expression compared with the solvent control, whereas lower doses of genistein (12.5 μM) promoted the expression of FAK and paxillin. Snail is an important transcription factor that regulates the expression of E-cadherin. It has been shown that loss of E-cadherin expression occurs frequently in malignant melanocytes [37]. Our results showed that high doses of genistein inhibited Snail expression, whereas low concentrations had the opposite effect. These results correlate with our previous observations showing that genistein inhibited EMT in dual ways (Figure 9).

## DISCUSSION

Genistein has been reported to suppress tumor cell growth and induce apoptosis in several tumor cell lines through different molecular pathways, including cell cycle arrest, mitochondria-dependent pathways, MAPK pathways, and caspase-dependent pathways [38–43]. However, little is currently known about genistein's effects on melanoma cells, especially its anti-metastatic potential on the FAK/paxillin pathway. Melanomas are skin tumors that generally have a poor cure rate because of their invasive behavior. Therefore, the present study was undertaken to elucidate the activity of genistein on B16F10 melanoma cells. Our experiments focused on the effects of genistein on cell growth, apoptosis, cell morphology, invasion, and migration of B16F10 melanoma cells *in vitro*.

In this study, genistein exerted both anti-proliferative and anti-metastatic effects on B16F10 melanoma cells. Furthermore, genistein induced its apoptotic effect in a dose-dependent manner. This observation is in agreement with the cytotoxic properties of genistein reported in other studies using different cell types [40, 43, 44].

The early stages of cell migration and invasion involve ruffling of the plasma membrane into distinct structures such as filopodia or lamellipodia, which contain bundles of actin filaments and associated proteins [3]. We observed that genistein leads to changes in cell morphology involving the formation of irregular shapes such as diamond shapes or slim pseudopodia-like protrusions. Our immunostaining results showed that genistein disrupted the structure of F-actin and reduced p-FAK and p-paxillin levels in focal adhesion contacts at the leading edges of B16F10 cells. The results indicate that genistein impairs the formation of focal adhesions and actin bundles via suppression of FAK and paxillin phosphorylation-induced activation. This result is consistent with a previous study in which blocking the expression or function of FAK and paxillin reduced cellular mobility [45].

To further investigate whether the morphological changes are related to invasion and migration, we first evaluated the adhesion (the first step in invasion and migration) of B16F10 cells after genistein treatment. Our results demonstrated that adhesion was significantly decreased. Moreover, genistein inhibited metastasis in a dose-dependent manner, and a high concentration of genistein inhibited cell migration and invasion. Interestingly, a lower dose of genistein promoted cell migration and invasion. Furthermore, our results demonstrated that treatment with 100 μM genistein strongly inhibited the phosphorylation of FAK and paxillin. The protein levels of α-actinin, vinculin, and tensin-2 were also strongly regulated by genistein in a concentration-dependent manner. Specifically, lower genistein doses (12.5–25 μM) increased, whereas a higher dose (50-100 μM) inhibited, protein expression. The results indicate that genistein influences both cell migration and invasion by regulating the FAK/paxillin pathway. Moreover, genistein regulates FAK and paxillin gene expression in dual ways. As previous studies reported, genistein influences cancer progression mainly by targeting the NF-κB, AKT, and caspase pathways [46]. Our results showed for the first time that genistein influences the FAK/paxillin pathway in melanomas. Further, Snail is an important transcription factor regulating the EMT of various cancer cells, and its overexpression in tumor tissues (various cancers including melanoma) is closely correlated with tumor progression (metastasis and recurrence) [47–49]. Our results showed that a high concentration of genistein significantly inhibits Snail expression, whereas a lower concentration enhances snail expression. These results are in agreement with our *in vitro* tests on both the migration and invasion of cells in the presence of genistein.

In addition, we analyzed the phosphorylation of the MAP kinases p38, ERK, and JNK in response to genistein treatment in B16F10 cells. Our results showed that the MAPK pathway in melanoma cells is highly activated without any treatment, whereas a high concentration of genistein strongly inhibited the phosphorylation of p38, ERK, and JNK. In contrast, lower concentrations of genistein stimulated the phosphorylation of these proteins. These results indicate that genistein exerts dual functions in regulating the MAPK cascade. It has been reported that genistein regulates the activation of MAPK in different cell types [50–52]. However, our results showed that genistein regulated MAPK in two different manners: stimulating the pathway at a lower concentration and inhibiting it at a higher concentration.

**Figure 10:**
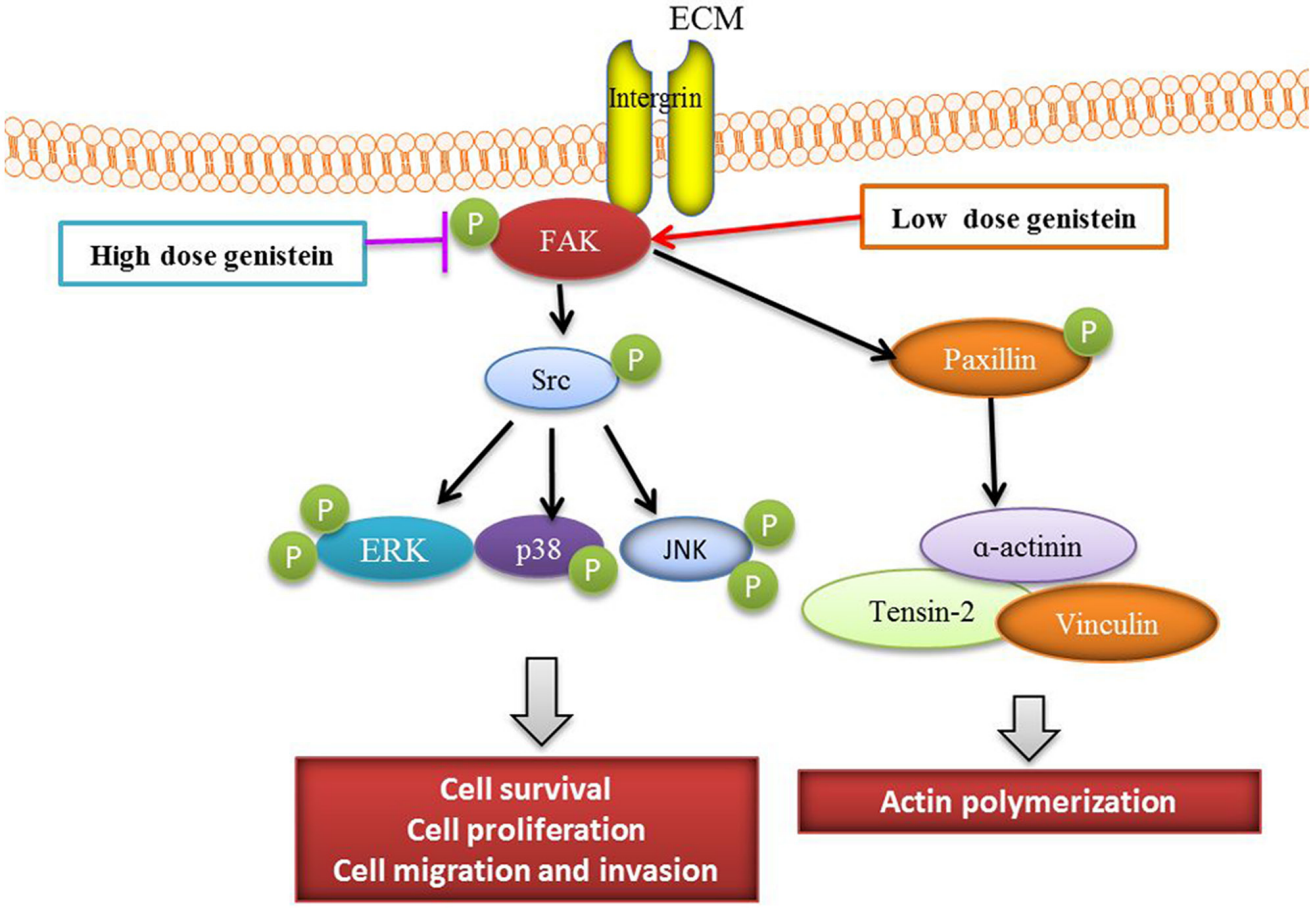
Scheme of the mechanism by which genistein affects melanoma cell survival, proliferation, and migration

In conclusion, genistein exerted dual functions in melanoma cells. At a higher dose, genistein decreased cell viability, induced apoptosis, and inhibited the adhesion, migration, and invasion of B16F10 cells by mediating the FAK/paxillin and MAPK pathways. At a lower dose, genistein promoted tumor progression via activation of the MAPK and FAK/paxillin signaling cascades. Collectively, the effect of genistein on melanoma is highly dependent on its concentration (Figure 10). Therefore, genistein should be used with caution as a potential chemotherapeutic candidate in future preclinical and clinical study.

## MATERIALS AND METHODS

### Materials

Dulbecco's modified Eagle's Medium (DMEM), 100× penicillin and streptomycin, and fetal bovine serum (FBS) were acquired from Lonza (Verviers, Belgium). The Annexin-V–PI Apoptosis Kit and TUNEL Apo-Green Detection Kit were purchased from Biotool (Shanghai, China). DMSO and genistein were both purchased from Sigma-Aldrich (Shanghai, China), and a stock solution (200 mM) of genistein was prepared in DMSO. Matrigel™ was acquired from Invitrogen (Karlsruhe, Germany), and WST-8 and the Phosphatase and Protease Inhibitor Cocktail Set I were acquired from Biotool. First and second antibodies were purchased from Cell Signaling Technology (Danvers, MA, USA). Detailed information regarding the antibodies we used are listed in Table 1. The enhanced chemiluminescence (ECL) detection system was obtained from Biotool.

**Table 1:** List of antibodies used in the experiments

	Name	Source	ID	Reference	Usage
**1**	Paxillin	Rabbit, pAb	#2542 Cell Signaling	[57, 58]	WB
**2**	Phospho-p44/42 MAPK (Thr202/Tyr204) (D13.14.4E)	Rabbit, mAb	#9910 Cell Signaling	[59]	WB
**3**	Phospho-p38 MAPK (Thr180/Tyr182) (D3F9)	Rabbit, mAb	#9910 Cell Signaling	[60]	WB
**4**	Phospho-SAPK/JNK (Thr183/Tyr185) (81E11)	Rabbit, mAb	#9910 Cell Signaling	[61]	WB
**5**	Anti-rabbit IgG, HRP-linked Antibody	Goat	#7074 Cell Signaling	[59]	WB
**6**	Phospho-Paxillin (Tyr118)	Rabbit, pAb	#2541 Cell Signaling	[58]	WB
**7**	p44/42MAP Kinase (137F5)	Rabbit, mAb	#9926 Cell Signaling	[59]	WB
**8**	p38 MAPK (D13E1)	Rabbit, mAb	#9926 Cell Signaling	[62]	WB
**9**	SAPK/JNK (56G8)	Rabbit, mAb	#9926 Cell Signaling	[60]	WB
**10**	α-Actinin (D6F6)	Rabbit, mAb	#13430 Cell Signaling	[63]	WB
**11**	FAK	Rabbit, pAb	#13430 Cell Signaling	[64]	WB
**12**	Tensin 2	Rabbit, pAb	#13430 Cell Signaling	[65]	WB
**13**	Vinculin	Rabbit, pAb	#13430 Cell Signaling	[66]	WB
**14**	Phospho-FAK (Tyr925)	Rabbit, pAb	#9330 Cell Signaling	[64]	WB
**15**	Vimentin (D21H3)	Rabbit, mAb	#9782 Cell Signaling	[67]	WB, IF
**16**	GAPDH-HRP	Mouse, mAb	#ab011 Multi Science		WB
**17**	Anti-FAK antibody [EP695Y]	Rabbit, mAb	#Ab40794 Abcam	[68]	IF
**18**	Anti-Paxillin antibody [Y113]	Rabbit, mAb	#Ab32084 Abcam	[69]	IF
**19**	Phospho-Paxillin (Tyr118) Antibody	Rabbit, pAb	#2541 Cell Signaling	[70]	IF
**20**	Anti-FAK (phospho Y925) antibody	Rabbit, pAb	# Ab38512 Abcam	[71]	IF
**21**	Anti-rabbit IgG (H+L), F(ab’)2 Fragment (Alexa Fluor® 555 Conjugate)	Goat	#4413 Cell Signaling	[72]	IF
**22**	CytoPainter Phalloidin-iFluor 488 Reagent		#Ab176753 Abcam	[73]	IF
**23**	DAPI staining buffer	#C1005 Beyotime	[74]	IF	

### Cell culture

The murine melanoma cell line B16F10 was purchased from the Type Culture Collection of the Chinese Academy of Sciences in Shanghai, China. The cells were grown in DMEM supplemented with 10% FBS at 37 °C in a 5% CO_2_ air atmosphere.

### Analysis of cell viability by WST-8 assay

B16F10 cells (100 μL) were seeded into 96-well cell culture plates at 5 × 10^5^/mL in DMEM containing various concentrations of genistein. Cells were incubated for 24 and 48 h under standard culture conditions. Subsequently, 10 μL of WST-8 were added to each well, and the plate was placed on a plate shaker for 1 min to ensure optimal mixing. After 1 h of incubation at 37 °C and 5% CO_2_, the OD at 450 nm was determined using a microtiter plate reader (BioRad, Hercules, CA, USA). For data analysis, the mean values and standard deviations were determined from three replicates.

### Cell counting

Cells (1 mL) were seeded into each well of 24-well cell culture plates at 5 × 10^5^/mL and incubated for 24 and 48 h with or without genistein. The cells were counted under a digital fluorescence microscope (Nikon, Tochigi, Japan).

### Colony formation assay

The colony formation assay was performed as reported previously [53]. Cells (1 × 10^3^/well) were plated in 6-well plates containing DMEM with 10% FBS at 37˚C. After 24 h, the cells were treated with different concentrations of genistein (0, 12.5, 25, 50, and 100 μM) for 24 h, and then cells were allowed to grow for 7 days in the absence of genistein in 6-well plates without a cushion of soft agar. Cells were fixed and stained with crystal violet (0.5%) for 20 min at room temperature. Images were captured using the inverted microscope (Nikon).

### Morphology

Cells (1 mL) were seeded into each well of 96-well cell culture plates at 5 × 10^5^/mL and incubated for 24 and 48 h with or without genistein. Pictures were taken using a digital fluorescence microscope equipped with an NIE-analysis soft image system (Nikon).

### Apoptosis assay

Cells were seeded into 6-well plates at a concentration of 5 × 10^5^/mL and allowed to settle for 2 h. Next, the indicated concentrations of genistein were added. After treatment for 24, 48 h, all cells (adherent and non-adherent) were collected. Annexin-V-(FITC) and propidium iodide were used for staining according to the manufacturer's instructions. Solvent-treated cells were used as the control. The double-stained cells were subsequently analyzed by the FACSAria™ flow cytometer (Becton, Dickinson and Company, Franklin Lakes, NJ, USA). All experiments were performed using three independent cell cultures. At least 10,000 cells were counted each time.

### Terminal deoxynucleotidyl transferase dUTP nick end labeling (TUNEL) assay

Coverslips were deposited into each well of a 24-well plate. Next, 1 mL of 5 × 10^5^ cells/mL were seeded into each well of 24-well cell culture plates and incubated for 24 and 48 h with or without genistein. The supernatant was removed, and the cells were washed twice with 750 μL phosphate buffer saline (PBS). After washing, the cells were fixed with 750 μL 4% paraformaldehyde (PFA) for 15 min at room temperature. Cells were then washed twice with PBS to remove the PFA. For permeabilization, cells were incubated with 750 μL 0.1% Triton X in PBS for 5 min. Cells were then washed three times with PBS. TUNEL Apo-Green buffer (100 μL) was added for 1 h. Cells were washed twice with PBS to remove unbound dye. Next, DAPI solution (100 μL) (Beyotime, Beijing, China) was added to stain the nuclei of the cells. The coverslips were removed carefully, and the samples were examined by digital fluorescence microscopy.

### Adhesion assay

An adhesion assay was performed as reported previously [54]. Cells were plated in a 12-well plate at a concentration of 5×10^5^/mL for 12 h and then incubated with different concentrations of genistein for 24 h. Cells were then harvested and re-seeded in a 24-well plate for 3 h. Cells were washed three times with PBS and fixed with 4% PFA at room temperature for 15 min. Cristal violet staining solution (Beyotime) was added to cells for 10 min at room temperature. The cells were then washed twice with PBS. The OD value was measured at 570 nm using a microplate reader (BioRad). Thepercentage of adhered cells was calculated relative to those in the control sample.

### Wound healing mobility assay

Cells were seeded at a concentration of 5×10^5^/well into a 6-well plate and grown to approximately 90% confluence after 24 h. The medium was removed, and cell monolayers were wounded by manually scraping the cells using a sterile P200 micropipette tip. Cellular debris was washed away three times with PBS, and cells were then cultured in serum-free medium containing different concentrations of genistein for 24 h under standard culture conditions. The wound areas were then photographed. Migration was quantified by measuring the distances from the wound edges, and the percentage of distance migrated was calculated relative to that of the control [55].

### Cell migration and invasion assay

Cells (4×10^4^/well) were placed in Transwell^®^ cell culture chambers (8 mm pore size; Corning, Lowell, MA, USA), which were coated with Matrigel™ for a 24-h invasion assay. The cell suspension was placed in the upper chamber of the Transwell^®^ insert and incubated with different concentrations of genistein and DMSO in serum-free DMEM. The lower chamber was filled with DMEM containing 20% FBS, which was used as a chemoattractant. Cells were incubated for 24 h and allowed to migrate or invade. Cells that migrated or invaded into the lower surface of the membrane were fixed with 4% PFA and then stained with crystal violet as described previously. Five random fields were counted per chamber using an inverted microscope. Each sample was assayed in triplicate as described previously [54].

### Western blot analysis

Western blot analysis was performed as described previously [50]. Cells were pretreated with or without DMSO (control) and genistein (12.5–100 μM) for 24 h. Cell lysates were prepared in ice-cold lysis buffer (MilliQ water + 100× Phosphatase and Protease Inhibitor Cocktail Set I). Proteins (40 μg/lane) were separated by gel electrophoresis on 10% SDS-PAGE gels. Separated proteins were transferred onto a PVDF membrane (BioRad) by electroblotting at 100 V for 90 min. Membranes were probed with the respective primary antibody followed by an HRP-conjugated secondary antibody and detected by ECL chemiluminescence. The primary antibodies were used at a 1:1000 dilution and secondary antibodies at a 1:3000 dilution.

### Immunofluorescence staining

An immunofluorescence assay was performed as reported previously [3, 56]. Briefly, cells were incubated for 24 h with or without 100 μM genistein. The cells were fixed in 4% PFA for 20 min at room temperature. Next, the cells were permeabilized with 0.1% Triton X-100 in PBS for 15 min and blocked with 2% bovine serum albumin (BSA) for 1 h. Subsequently, the cells were washed with PBS and BSA prior to incubating with anti-vimentin (1:500), anti-p-paxillin (1:50), anti-paxillin (1:500), anti-p-FAK (1:500), or anti-FAK (1:500) primary antibodies overnight at 4°C. Samples were then washed five times with PBS, followed by incubation with anti-rabbit IgG secondary antibodies (1:500) and iFluor 488-conjugated phalloidin (1:1000) (Abcam, Shanghai, China) for 1 h at room temperature. Next, 100 μL DAPI solution (Beyotime) were added to stain the nuclei of the cells. Samples were then examined using the TCS SP8 STED ultra high resolution laser confocal microscopy (Leica, Wetzlar, Germany).

### Real-time PCR

qPCR was performed as reported previously [24]. Briefly, cells were pretreated with or without DMSO (control) and genistein (12.5–100 μM) for 5 h, and total RNA was extracted using the RNeasy Plus Mini Kit (Qiagen, Valencia, CA, USA). cDNA was prepared using a reverse transcription reagent kit (Selleck Company, Shanghai, China) according to the manufacturer's instructions. Target genes were amplified using the Light Cycler^®^ 96 Real-Time PCR System (Roche, Indianapolis, IN, USA) with the following specific primers:

FAK, 5′-CCATGCCCTCGAAAAGCTATG-3′ (forward) and 5′- TGACGCATTGTTAAGGCTTCT-3′ (reverse);

Paxillin, 5′-GGCATCCCAGAAAATAACACTC C-3′ (forward) and 5′-GCCCTGCATCTTGAAATC TGA-3′ (reverse);

Snail, 5′-GGTCCCCAACTACGGGAAAC-3′ (forward) and 5′- CTGTAGGGGGTCACTGGGATT-3′ (reverse);

Vimentin, 5′-CGTCCACACGCACCTACAG-3′ (forward) and 5′- GGGGGATGAGGAATAGAGGCT-3′ (reverse);

GAPDH, 5′-GCACCGTCAAGGCTGAGAAC-3′ (forward) and 5′-TGGTGAAGACGCCAGTGGA-3′ (reverse).

GAPDH was utilized for normalization. Each sample was tested in triplicate, and the relative gene levels were normalized using the 2^−ΔΔCt^ method.

### Statistical analysis

All data are presented as means ± S.D from at least three experiments. Statistical significance was determined using the Student's t-test, with p<0.05 considered statistically significant.

## References

[1] Ascierto PA, Bastholt L, Hersey P, Cinat G, Eggermont AM, Hauschild A, Espinosa E, Robert C (2015). Side effects and toxicities of targeted therapies in stage IV melanoma. American journal of therapeutics.

[2] Atkinson V (2015). Medical management of malignant melanoma. Australian prescriber.

[3] Chiu KY, Wu CC, Chia CH, Hsu SL, Tzeng YM (2016). Inhibition of growth, migration and invasion of human bladder cancer cells by antrocin, a sesquiterpene lactone isolated from Antrodia cinnamomea, and its molecular mechanisms. Cancer letters.

[4] Zhao X, Guan JL (2011). Focal adhesion kinase and its signaling pathways in cell migration and angiogenesis. Advanced drug delivery reviews.

[5] Hood JD, Cheresh DA (2002). Role of integrins in cell invasion and migration. Nature reviews Cancer.

[6] McCawley LJ, Matrisian LM (2000). Matrix metalloproteinases: multifunctional contributors to tumor progression. Molecular medicine today.

[7] Yamaguchi H, Wyckoff J, Condeelis J (2005). Cell migration in tumors. Current opinion in cell biology.

[8] Friedl P, Wolf K (2003). Tumour-cell invasion and migration: diversity and escape mechanisms. Nature reviews Cancer.

[9] Nabeshima K, Inoue T, Shimao Y, Sameshima T (2002). Matrix metalloproteinases in tumor invasion: role for cell migration. Pathology international.

[10] Zhang J, Hochwald SN (2014). The role of FAK in tumor metabolism and therapy. Pharmacology & therapeutics.

[11] Schaller MD (2001). Biochemical signals and biological responses elicited by the focal adhesion kinase. Biochimica et biophysica acta.

[12] Siesser PM, Hanks SK (2006). The signaling and biological implications of FAK overexpression in cancer. Clinical cancer research.

[13] Ucar DA, Hochwald SN (2010). FAK and interacting proteins as therapeutic targets in pancreatic cancer. Anti-cancer agents in medicinal chemistry.

[14] Sulzmaier FJ, Jean C, Schlaepfer DD (2014). FAK in cancer: mechanistic findings and clinical applications. Nature reviews Cancer.

[15] Bolos V, Gasent JM, Lopez-Tarruella S, Grande E (2010). The dual kinase complex FAK-Src as a promising therapeutic target in cancer. OncoTargets and therapy.

[16] Golubovskaya VM (2014). Targeting FAK in human cancer: from finding to first clinical trials. Frontiers in bioscience.

[17] Dunn KB, Heffler M, Golubovskaya VM (2010). Evolving therapies and FAK inhibitors for the treatment of cancer. Anti-cancer agents in medicinal chemistry.

[18] Schlaepfer DD, Hauck CR, Sieg DJ (1999). Signaling through focal adhesion kinase. Progress in biophysics and molecular biology.

[19] Subauste MC, Pertz O, Adamson ED, Turner CE, Junger S, Hahn KM (2004). Vinculin modulation of paxillin-FAK interactions regulates ERK to control survival and motility. The Journal of cell biology.

[20] Webb DJ, Donais K, Whitmore LA, Thomas SM, Turner CE, Parsons JT, Horwitz AF (2004). FAK-Src signalling through paxillin, ERK and MLCK regulates adhesion disassembly. Nature cell biology.

[21] Du T, Qu Y, Li J, Li H, Su L, Zhou Q, Yan M, Li C, Zhu Z, Liu B (2014). Maternal embryonic leucine zipper kinase enhances gastric cancer progression via the FAK/Paxillin pathway. Molecular cancer.

[22] Deramaudt TB, Dujardin D, Noulet F, Martin S, Vauchelles R, Takeda K, Ronde P (2014). Altering FAK-paxillin interactions reduces adhesion, migration and invasion processes. PloS one.

[23] Chen JY, Tang YA, Huang SM, Juan HF, Wu LW, Sun YC, Wang SC, Wu KW, Balraj G, Chang TT, Li WS, Cheng HC, Wang YC (2011). A novel sialyltransferase inhibitor suppresses FAK/paxillin signaling and cancer angiogenesis and metastasis pathways. Cancer research.

[24] Fan T, Chen J, Zhang L, Gao P, Hui Y, Xu P, Zhang X, Liu H (2016). Bit1 knockdown contributes to growth suppression as well as the decreases of migration and invasion abilities in esophageal squamous cell carcinoma via suppressing FAK-paxillin pathway. Molecular cancer.

[25] Kanteti R, Batra SK, Lennon FE, Salgia R (2016). FAK and paxillin, two potential targets in pancreatic cancer. Oncotarget.

[26] Schaller MD (2004). FAK and paxillin: regulators of N-cadherin adhesion and inhibitors of cell migration?. The Journal of cell biology.

[27] Panetti TS (2002). Tyrosine phosphorylation of paxillin, FAK, and p130CAS: effects on cell spreading and migration. Frontiers in bioscience.

[28] Grimaldi AM, Simeone E, Festino L, Vanella V, Palla M, Ascierto PA (2015). Novel mechanisms and therapeutic approaches in melanoma: targeting the MAPK pathway. Discovery medicine.

[29] Cheng Y, Zhang G, Li G (2013). Targeting MAPK pathway in melanoma therapy. Cancer metastasis reviews.

[30] Thiery JP (2003). Epithelial-mesenchymal transitions in development and pathologies. Current opinion in cell biology.

[31] Peinado H, Olmeda D, Snail Cano A (2007). Zeb and bHLH factors in tumour progression: an alliance against the epithelial phenotype?. Nature reviews Cancer.

[32] Radzikowski C, Wietrzyk J, Grynkiewicz G, Opolski A (2004). Genistein: a soy isoflavone revealing a pleiotropic mechanism of action - clinical implications in the treatment and prevention of cancer [Article in Polish]. Postepy higieny i medycyny doswiadczalnej.

[33] Russo M, Russo GL, Daglia M, Kasi PD, Ravi S, Nabavi SF, Nabavi SM (2016). Understanding genistein in cancer: The “good” and the “bad” effects: A review. Food chemistry.

[34] Spagnuolo C, Russo GL, Orhan IE, Habtemariam S, Daglia M, Sureda A, Nabavi SF, Devi KP, Loizzo MR, Tundis R, Nabavi SM (2015). Genistein and cancer: current status, challenges, and future directions. Advances in nutrition.

[35] Pavese JM, Farmer RL, Bergan RC (2010). Inhibition of cancer cell invasion and metastasis by genistein. Cancer metastasis reviews.

[36] Carlino MS, Long GV, Kefford RF, Rizos H (2015). Targeting oncogenic BRAF and aberrant MAPK activation in the treatment of cutaneous melanoma. Critical reviews in oncology/hematology.

[37] Poser I, Dominguez D, de Herreros AG, Varnai A, Buettner R, Bosserhoff AK (2001). Loss of E-cadherin expression in melanoma cells involves up-regulation of the transcriptional repressor Snail. The Journal of biological chemistry.

[38] Kim EJ, Shin HK, Park JH (2005). Genistein inhibits insulin-like growth factor-I receptor signaling in HT-29 human colon cancer cells: a possible mechanism of the growth inhibitory effect of Genistein. Journal of medicinal food.

[39] Yu Z, Li W, Liu F (2004). Inhibition of proliferation and induction of apoptosis by genistein in colon cancer HT-29 cells. Cancer letters.

[40] Qin J, Teng J, Zhu Z, Chen J, Huang WJ (2016). Genistein induces activation of the mitochondrial apoptosis pathway by inhibiting phosphorylation of Akt in colorectal cancer cells. Pharmaceutical biology.

[41] Lee YK, Park OJ (2013). Soybean isoflavone genistein regulates apoptosis through NF-kappaB dependent and independent pathways. Experimental and toxicologic pathology.

[42] Li Z, Li J, Mo B, Hu C, Liu H, Qi H, Wang X, Xu J (2008). Genistein induces cell apoptosis in MDA-MB-231 breast cancer cells via the mitogen-activated protein kinase pathway. Toxicol In Vitro.

[43] Dhandayuthapani S, Marimuthu P, Hormann V, Kumi-Diaka J, Rathinavelu A (2013). Induction of apoptosis in HeLa cells via caspase activation by resveratrol and genistein. J Med Food.

[44] Xia J, Cheng L, Mei C, Ma J, Shi Y, Zeng F, Wang Z, Wang Z (2014). Genistein inhibits cell growth and invasion through regulation of miR-27a in pancreatic cancer cells. Current pharmaceutical design.

[45] Hiscox S, Barnfather P, Hayes E, Bramble P, Christensen J, Nicholson RI, Barrett-Lee P (2011). Inhibition of focal adhesion kinase suppresses the adverse phenotype of endocrine-resistant breast cancer cells and improves endocrine response in endocrine-sensitive cells. Breast cancer research and treatment.

[46] Banerjee S, Li Y, Wang Z, Sarkar FH (2008). Multi-targeted therapy of cancer by genistein. Cancer letters.

[47] Kuphal S, Palm HG, Poser I, Bosserhoff AK (2005). Snail-regulated genes in malignant melanoma. Melanoma research.

[48] Becker KF, Rosivatz E, Blechschmidt K, Kremmer E, Sarbia M, Hofler H (2007). Analysis of the E-cadherin repressor Snail in primary human cancers Cells, tissues, organs.

[49] Stasiak M, Boncela J, Perreau C, Karamanou K, Chatron-Colliet A, Proult I, Przygodzka P, Chakravarti S, Maquart FX, Kowalska MA, Wegrowski Y, Brezillon S (2016). Lumican inhibits SNAIL-induced melanoma cell migration specifically by blocking MMP-14 activity. PloS one.

[50] Cui S, Wienhoefer N, Bilitewski U (2014). Genistein induces morphology change and G2/M cell cycle arrest by inducing p38 MAPK activation in macrophages. International immunopharmacology.

[51] Xu L, Bergan RC (2006). Genistein inhibits matrix metalloproteinase type 2 activation and prostate cancer cell invasion by blocking the transforming growth factor beta-mediated activation of mitogen-activated protein kinase-activated protein kinase 2-27-kDa heat shock protein pathway. Molecular pharmacology.

[52] Huang X, Chen S, Xu L, Liu Y, Deb DK, Platanias LC, Bergan RC (2005). Genistein inhibits p38 map kinase activation, matrix metalloproteinase type 2, and cell invasion in human prostate epithelial cells. Cancer research.

[53] Shan X, Aziz F, Tian LL, Wang XQ, Yan Q, Liu JW (2015). Ginsenoside Rg3-induced EGFR/MAPK pathway deactivation inhibits melanoma cell proliferation by decreasing FUT4/LeY expression. International journal of oncology.

[54] Shiue YW, Lu CC, Hsiao YP, Liao CL, Lin JP, Lai KC, Yu CC, Huang YP, Ho HC, Chung JG (2016). Casticin induced apoptosis in A375.S2 human melanoma cells through the inhibition of NF-kappaB and mitochondria-dependent pathways in vitro and inhibited human melanoma xenografts in a mouse model in vivo. The American journal of Chinese medicine.

[55] Yeh PS, Wang W, Chang YA, Lin CJ, Wang JJ, Chen RM (2016). Honokiol induces autophagy of neuroblastoma cells through activating the PI3K/Akt/mTOR and endoplasmic reticular stress/ERK1/2 signaling pathways and suppressing cell migration. Cancer letters.

[56] Jiang S, Gao Y, Hou W, Liu R, Qi X, Xu X, Li J, Bao Y, Zheng H, Hua B (2016). Sinomenine inhibits A549 human lung cancer cell invasion by mediating the STAT3 signaling pathway. Oncology letters.

[57] Bellis SL, Perrotta JA, Curtis MS, Turner CE (1997). Adhesion of fibroblasts to fibronectin stimulates both serine and tyrosine phosphorylation of paxillin. The Biochemical journal.

[58] Turner CE (2000). Paxillin and focal adhesion signalling. Nature cell biology.

[59] Lewis TS, Shapiro PS, Ahn NG (1998). Signal transduction through MAP kinase cascades. Advances in cancer research.

[60] Schaeffer HJ, Weber MJ (1999). Mitogen-activated protein kinases: specific messages from ubiquitous messengers. Molecular and cellular biology.

[61] Whitmarsh AJ, Davis RJ (1998). Structural organization of MAP-kinase signaling modules by scaffold proteins in yeast and mammals. Trends in biochemical sciences.

[62] Garrington TP, Johnson GL (1999). Organization and regulation of mitogen-activated protein kinase signaling pathways. Current opinion in cell biology.

[63] Otey CA, Carpen O (2004). Alpha-actinin revisited: a fresh look at an old player. Cell motility and the cytoskeleton.

[64] Parsons JT, Martin KH, Slack JK, Taylor JM, Weed SA (2000). Focal adhesion kinase: a regulator of focal adhesion dynamics and cell movement. Oncogene.

[65] Clark K, Howe JD, Pullar CE, Green JA, Artym VV, Yamada KM, Critchley DR (2010). Tensin 2 modulates cell contractility in 3D collagen gels through the RhoGAP DLC1. Journal of cellular biochemistry.

[66] Izard T, Evans G, Borgon RA, Rush CL, Bricogne G, Bois PR (2004). Vinculin activation by talin through helical bundle conversion. Nature.

[67] Helfand BT, Chang L, Goldman RD (2004). Intermediate filaments are dynamic and motile elements of cellular architecture. Journal of cell science.

[68] Dave JM, Abbey CA, Duran CL, Seo H, Johnson GA, Bayless KJ (2016). Hic-5 mediates the initiation of endothelial sprouting by regulating a key surface metalloproteinase. Journal of cell science.

[69] Izumi D, Ishimoto T, Miyake K, Sugihara H, Eto K, Sawayama H, Yasuda T, Kiyozumi Y, Kaida T, Kurashige J, Imamura Y, Hiyoshi Y, Iwatsuki M (2016). CXCL12/CXCR4 activation by cancer-associated fibroblasts promotes integrin beta1 clustering and invasiveness in gastric cancer. International journal of cancer.

[70] Muralidharan AR, Maddala R, Skiba NP, Rao PV (2016). Growth Differentiation Factor-15-Induced Contractile Activity and Extracellular Matrix Production in Human Trabecular Meshwork Cells. Investigative ophthalmology & visual science.

[71] Tomioka H, Nakagami H, Tenma A, Saito Y, Kaga T, Kanamori T, Tamura N, Tomono K, Kaneda Y, Morishita R (2014). Novel anti-microbial peptide SR-0379 accelerates wound healing via the PI3 kinase/Akt/mTOR pathway. PloS one.

[72] Qiao Y, Qian Y, Wang J, Tang X (2016). Melanoma cell adhesion molecule stimulates yes-associated protein transcription by enhancing CREB activity via c-Jun/c-Fos in hepatocellular carcinoma cells. Oncology letters.

[73] Kim D, Lim JY, Kwon S (2016). Development of Vibrational Culture Model Mimicking Vocal Fold Tissues. Annals of biomedical engineering.

[74] Zhou H, Qian J, Wang J, Yao W, Liu C, Chen J, Cao X (2009). Enhanced bioactivity of bone morphogenetic protein-2 with low dose of 2-N, 6-O-sulfated chitosan in vitro and in vivo. Biomaterials.

